# The Use of the Hypotension Prediction Index Integrated in an Algorithm of Goal Directed Hemodynamic Treatment during Moderate and High-Risk Surgery

**DOI:** 10.3390/jcm10245884

**Published:** 2021-12-15

**Authors:** Marina Tsoumpa, Aikaterini Kyttari, Stamo Matiatou, Maria Tzoufi, Panayota Griva, Emmanouil Pikoulis, Maria Riga, Paraskevi Matsota, Tatiana Sidiropoulou

**Affiliations:** 1 Second Department of Anesthesiology, Attikon University Hospital, National and Kapodistrian University of Athens, 12461 Athens, Greece; tsoumpa.marina@gmail.com (M.T.); akyttari@gmail.com (A.K.); stamo.matiatou@gmail.com (S.M.); majtzoufi@yahoo.com (M.T.); nagriva@gmail.com (P.G.); mriga83@gmail.com (M.R.); matsota@yahoo.gr (P.M.); 2 Third Department of Surgery, Attikon University Hospital, National and Kapodistrian University of Athens, 12461 Athens, Greece; mpikoul@med.uoa.gr

**Keywords:** intraoperative hypotension, machine learning, Hypotension Prediction Index, non-cardiac surgery, goal directed hemodynamic therapy

## Abstract

(1) Background: The Hypotension Prediction Index (HPI) is an algorithm that predicts hypotension, defined as mean arterial pressure (MAP) less than 65 mmHg for at least 1 min, based on arterial waveform features. We tested the hypothesis that the use of this index reduces the duration and severity of hypotension during noncardiac surgery. (2) Methods: We enrolled adults having moderate- or high-risk noncardiac surgery with invasive arterial pressure monitoring. Participating patients were randomized 1:1 to standard of care or hemodynamic management with HPI guidance with a goal directed hemodynamic treatment protocol. The trigger to initiate treatment (with fluids, vasopressors, or inotropes) was a value of HPI of 85 (range, 0–100) or higher in the intervention group. Primary outcome was the amount of hypotension, defined as time-weighted average (TWA) MAP less than 65 mmHg. Secondary outcomes were time spent in hypertension defined as MAP more than 100 mmHg for at least 1 min; medication and fluids administered and postoperative complications. (3) Results: We obtained data from 99 patients. The median (IQR) TWA of hypotension was 0.16 mmHg (IQR, 0.01–0.32 mmHg) in the intervention group versus 0.50 mmHg (IQR, 0.11–0.97 mmHg) in the control group, for a median difference of −0.28 (95% CI, −0.48 to −0.09 mmHg; *p* = 0.0003). We also observed an increase in hypertension in the intervention group as well as a higher weight-adjusted administration of phenylephrine in the intervention group. (4) Conclusions: In this single-center prospective study of patients undergoing elective noncardiac surgery, the use of this prediction model resulted in less intraoperative hypotension compared with standard care. An increase in the time spent in hypertension in the treatment group was also observed, probably as a result of overtreatment. This should provide an insight for refining the use of this prediction index in future studies to avoid excessive correction of blood pressure.

## 1. Introduction

Intraoperative hypotension (IOH), due to anesthetic drugs, preoperative use of medications, concurrent diseases, or surgical maneuvers remains a major problem in patients undergoing general anesthesia [1]. According to various studies, patients undergoing non cardiac surgery have a 65–90% probability of experiencing one or more hypotensive episodes [2,3]. In large cohort studies, IOH has been associated with worse outcomes such as myocardial ischemia, renal injury and increased mortality [4,5,6,7,8,9,10,11]. It has been suggested that the increase in organ dysfunction observed with intraoperative hypotension is time dependent [12]. As a result, a decrease in both severity and duration of hypotensive episodes would, presumably, reduce organ injury. Although there is vast heterogeneity in the different definitions of IOH in the literature [2], according to the Perioperative Quality Initiative consensus statement (POQI-3), “Intraoperative mean arterial blood pressures below 60–70 mmHg are associated with myocardial injury, acute kidney injury, and death. Injury is a function of hypotension severity and duration” [13].

Recently the Hypotension Prediction Index (HPI) has been developed [3,14] by Edwards Lifesciences (Irvine, CA, USA) integrated into their EV1000 clinical platform. The software was developed as a predictive model using arterial waveform features. HPI can be used to predict a hypotensive episode, defined as mean arterial pressure (MAP) less than 65 mmHg for 1 min, at least 5 min before it occurs. This system was validated both internally and externally showing a sensitivity and specificity of approximately 85% [3]. The index ranges from 0 to 100, with higher figures implying higher hypotension probability.

In this single center prospective study, we aimed to validate the hypothesis that the use of the Hypotension Prediction Index integrated with an hemodynamic management protocol would decrease the amount of hypotension (MAP < 65 mmHg) during moderate or high risk noncardiac surgery.

## 2. Materials and Methods

### 2.1. Participants and Eligibility Criteria

The trial was conducted at the “Attikon University Hospital” in Athens, Greece. The trial was approved by the Institutional Scientific Review Board (639/639/25-09-2018) and was also retrospectively registered in ClinicalTrials.gov (accessed on 17 November 2021) (NCT04803903). Written consent was obtained from all the participants the day before surgery. The trial included 100 patients aged 18 or older undergoing elective non-cardiac surgery that required a minimum duration of 2 h under general anesthesia, with need for continuous invasive blood pressure monitoring intraoperatively. The aim was to maintain mean arterial pressure (MAP) above 65 mmHg.

The exclusion criteria included a MAP target other than 65 mmHg according to the attending anesthesiologist. Patients with significant hypotension as measured before surgery, with known left or right cardiac failure, known arrythmias (e.g., atrial fibrillation), cardiac shunts, severe aortic stenosis or the need for dialysis were also excluded. If surgery included clamping of the aorta or Pringle’s maneuver, the patient was not eligible for the trial. Finally, emergency procedures were also excluded from the trial.

### 2.2. Study Design

Prior to surgery, patients were randomly assigned into two groups with a 1:1 allocation ratio: the HPI (HPI-guided) group and the control (HPI-unguided) group. Group allocation was concealed from patients. Besides the standard operating room monitoring, which included electrocardiogram, pulse oximetry and end tidal carbon dioxide measurement, an arterial line (radial artery) was placed on each trial participant, after which the Acumen Flo-Traq transducer (Edwards LifeSciences, Irvine, CA, USA) was connected to an EV-1000 monitor (Edwards LifeSciences, Irvine, CA, USA) with the HPI software incorporated, and to a standard anesthesia machine monitor displaying the arterial pressure waveform. Use of the EV-1000 monitor was additional to standard care monitoring and available for consultation only to the intervention group. The index, displayed in the EV-1000 monitor, ranges between 0 and 100. When the index reaches 85, the EV-1000 monitor alerts the operator, and a secondary screen can be revealed. The secondary screen displays hemodynamic variables (heart rate, cardiac output (CO), cardiac index (CI), maximal rise of pressure over time (dP/dtmax), stroke volume (SV), stroke volume variation (SVV), dynamic arterial elastance (Eadyn), and systemic vascular resistance (SVR)) that provide information about the underlying cause of the predicted hypotension. All these parameters are updated every 20 s, as well as the HPI. All arterial lines were placed before induction of anesthesia. The quality of the arterial waveform signal was checked continuously by the attending anesthesiologist throughout the study.

**Figure 1:**
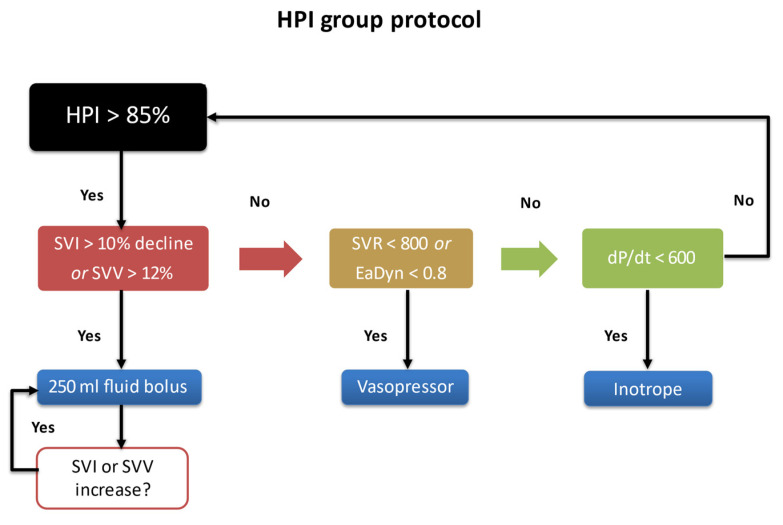
Goal directed hemodynamic protocol.

Attending anesthesiologists were informed about the study protocol and use of the HPI prior to initiation of the procedure. Intraoperatively, an observer was present to record surgery- and anesthesia-related details. In the intervention arm, when the HPI was above 85, the attending anesthesiologist was instructed as per the study protocol to act upon this immediately based on our study algorithm (Figure 1). Use of the study treatment algorithm ensured that the anesthesiologist had to think about the underlying cause and act accordingly. The study treatment algorithm was inspired by Pinsky [15].

Therefore, if the stroke volume index (SVI) showed more than 10% decline from baseline or the SVV indicated a greater that 12% increase, a bolus of 250 mL crystalloids was given according to the algorithm and was repeated after 5 min from the end of infusion if the SVV did not fall below 12% or the SVI increased by less than 10% of the baseline value. A value of Eadyn less than 0.8 or SVR less than 800, was an indication to give vasopressors and finally, if the dP/dt was less than 600, this was an indication of low contractility, hence, an inotrope was given. There were no prespecified indications on the choice or dosage for vasopressors or inοtropic drugs. SVI baseline values were measured before anesthesia induction.

In the control group, conventional treatment with invasive blood pressure monitoring was followed. Administration of fluids, vasopressors or inotropes were guided by hemodynamic parameters displayed on the standard anesthesia machine monitor at the discretion of the attending physician while the EV1000 monitor was blinded, and all sound alarms were silenced. Attending anesthesiologists were instructed to avoid MAP less than 65 mmHg.

After induction of anesthesia, all patients were ventilated with an 8 mL/kg tidal volume to ensure correct measuring of the hemodynamic parameters [16].

### 2.3. Outcomes

The primary outcome of the trial was the time-weighted average (TWA) spent in hypotension. A hypotensive event was defined per protocol as MAP less than 65 mmHg that lasted for at least 1 min. The hypotensive event ended when the MAP value was normalized. The time-weighted average is calculated as follows: area under the threshold divided by the total duration of surgery. The complete formula would be: TWA = (depth of hypotension (mmHg) below a MAP of 65 mmHg × time (minutes) spent below a MAP of 65 mmHg) ÷ total duration of surgery (minutes). The units for area under the threshold are mmHg × minutes and the units for TWA are mmHg.

Secondary outcomes included the incidence of intraoperative hypotension, defined as the number of hypotensive events, total time (in minutes) spent with hypotension and the percentage of time spent with hypotension during surgery. In order to estimate the possibility of overtreating patients, we also investigated, in a post hoc analysis, the time weighted average in hypertension. Hypertension was defined as a MAP above 100 mmHg for at least 1 min. Incidence of intraoperative hypertension (number of hypertensive events per patient) was also measured, time spent with hypertension as well as the percentage of time with hypertension.

Intraoperative exploratory variables registered in all patients were the amount of intraoperative crystalloids and colloids, amount of erythrocyte transfusion, cumulative dose of vasoactive medications (vasopressor = phenylephrine; inotrope = ephedrine), cumulative dose of anesthetics and analgesics, blood loss, and urine output. Postoperatively we recorded any morbidity and mortality, as well as length of stay in the intensive care unit and hospital. Morbidity was divided into cardiac (arrhythmias, myocardial infarction, and left ventricular failure), pulmonary (pneumonia, pulmonary edema, pneumothorax, need for mechanical ventilation or reintubation), renal (according to the AKIN criteria), and surgical complications.

### 2.4. Sample Size Calculation

The HPI is an innovative software and time-weighted average (TWA) is a relatively novel end point, therefore calculation of the sample size was based on the previously published literature [17]. We decided a priori that a 50% reduction in TWA in hypotension would be clinically meaningful. Prior to study initiation we calculated our hospitals’ average TWA of MAP < 65 mmHg, which was 0.54 with a standard deviation of 0.35. These results gave us an effect size of 0.77. Based on this, we considered that a sample size of 45 patients in each group would be required to detect this effect using a 2-group *t* test with an α = 0.05, a 2-sided significance level and 95% power. We increased the sample size to 50 to allow for dropouts. Sample size calculation was performed with G* Power software v3.1.1 (Universitat Kiel, Kiel, Germany).

### 2.5. Statistical Analysis

Normality was tested using the Shapiro–Wilk test. Comparisons between the two groups were performed using the Student’s *t*-test or the Mann–Whitney U test for continuous variables and the *χ*^2^ test for categorical variables. Median differences and the respective 95% confidence intervals were calculated with the Hodges–Lehmann method. Continuous data are reported as mean ± standard deviation (SD) or as median [interquartile range]; categorical data are reported as numbers (percentages). A *p*-value of 0.05 or less was considered statistically significant. Statistical analysis was carried out with the SPSS v23.0 software (SPSS Inc., Chicago, IL, USA) and SAS software, v9.4 (SAS Institute Inc., Cary, NC, USA).

## 3. Results

### 3.1. Study Population

**Figure 2:**
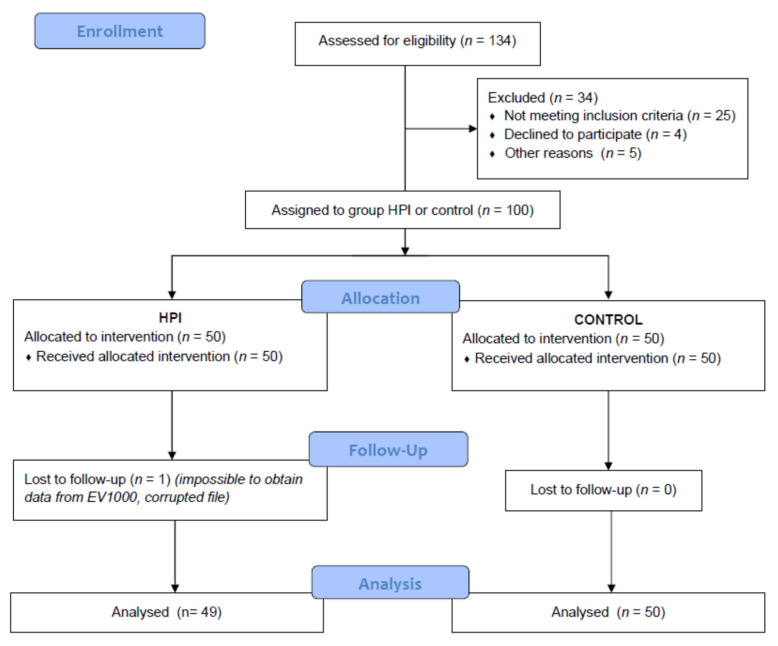
Participant flow diagram.

The study flow diagram is illustrated in Figure 2. From 5 November 2018 to 30 March 2021, 134 patients scheduled for surgery with general anesthesia were assessed for eligibility, and 25 patients who did not meet the inclusion criteria, were excluded from the study. Five more patients refused enrollment in the study, while 4 were not enrolled due to unavailability of the Flo-Traq transducer in our hospital. Therefore, a total of 100 patients were enrolled and assigned into two study groups. Data were analyzed from 99 patients because we were unable to obtain the file of one patient from the EV-1000 monitor due to data corruption. Table 1 shows the baseline characteristics of the patients included in the study. The majority of patients underwent gastrointestinal and hepatobiliary surgery.

### 3.2. Primary and Secondary Outcomes

The median time weighted average of hypotension was 0.16 mmHg (IQR, 0.01–0.32 mmHg) in the intervention group vs. 0.50 mmHg (IQR, 0.11–0.97 mmHg) in the control group, for a median difference of −0.28 (95% CI, −0.48 to −0.09 mmHg; *p* = 0.0003) (Table 2).

The median incidence of hypotension was 26 (IQR, 6–54) hypotensive episodes in the intervention group vs. 73 (IQR, 32–190) in the control group, for a median difference of −43 (95% CI, −78 to −18) episodes (*p* = 0.0002). The median incidence of hypotension was calculated including patients who had 0 hypotensive episodes.

The median total time of hypotension per patient was 8.68 (IQR, 2–17.3) minutes in the intervention group vs. 24.16 (IQR, 10.67–63.02) minutes in the control group, for a median difference of −14.33 (95% CI −26 to −6; *p* = 0.0002) minutes. Percentage of time in hypotension (time in hypotension/total monitoring time) was accordingly 3.7% (IQR, 0.9–6.3) for the intervention group and 10.2% (IQR, 3.4–16.8), for a median difference of −6.7 (95% CI, −9.6 to −3.4; *p* < 0.0001)

The median time weighted average of hypertension was 0.95 mmHg (IQR, 0.27–1.81 mmHg) in the intervention group vs. 0.29 mmHg (IQR, 0.08–0.83 mmHg) in the control group, for a median difference of 0.40 (95% CI, 0.10 to 0.83 mmHg; *p* = 0.0032) (Table 2).

The median incidence of hypertension was 73 (IQR, 40–115) hypertensive episodes in the intervention group vs. 34 (IQR, 14–65) in the control group, for a median difference of 29 (95% CI 11 to 51) episodes (*p* = 0.0022).

The median total time of hypertension per patient was 24.3 (IQR, 13.3–38) minutes in the intervention group vs. 11.33 (IQR, 4.67–21.67) minutes in the control group, for a median difference of 9.67 (95% CI 3.6 to 16.7, *p* = 0.0024) minutes. Percentage of time in hypertension (time in hypertension/total monitoring time) was 9.7% (IQR, 3.9–18) for the intervention group and 4.1% (IQR, 1.8–9.4), for a median difference of 3.8 (95% CI, 1.2 to 7.3, *p* = 0.0034). Average hypertension values were 109.4 mmHg (IQR, 105.6–112.4) for the intervention group and 106.9 mmHg (IQR, 103.6–110.7) for the control group for a median difference of 2.1 (95% CI, 0.01 to 4.43, *p* = 0.0438).

Two patients in each group did not receive vasopressors. Additionally, 2 patients in the HPI group and 3 in the control group received less than 0.2 mg of phenylephrine. Fourteen patients in the HPI group did not experience any hypotensive episodes versus four patients in the control group. Conversely, ten patients in the control group did not experience any hypertensive episodes versus four patients in the HPI group.

The median monitoring time was 255.33 (IQR, 181–295.98) in the intervention group and 242.33 (IQR, 169.27–375.18) in the control group. Adherence to the treatment algorithm of the intervention group was very high. In fact, 91% of alerts for an HPI >85% in the EV1000 monitor were followed by intervention. The remaining 9% of the HPI alarms that were not followed by intervention were due mostly to: (a) treatment had already begun before the alarm, (b) alarm fatigue leading to the physician ignoring the alarm, and (c) there was a surgical reason for a brief period of hypotension.

### 3.3. Drug Use and Adverse Events

There was no difference in crystalloid or colloid infusion between the two groups. Phenylephrine was the vasopressor used by all physicians. Cumulative phenylephrine use was not different between groups, but when adjusted with the patients’ weight, there was a significant difference between the HPI and the control group 0.43 (IQR, 0.16–0.67) vs. 0.27 (IQR, 0.06–0.6), respectively (*p* = 0.029). Anesthetic drug use did not differ among groups (Table 3).

### 3.4. Adverse Events

There was no difference in blood loss or urine output between groups. Length of stay in the hospital was also similar between groups.

Complications were similar between groups. There was one death registered per group due to surgical complications (intestinal ischemia and portal vein thrombosis) (Table 3).

## 4. Discussion

In this trial of 99 patients undergoing moderate to high-risk non-cardiac surgery, we found a significant, about 3-fold, decrease in time weighted average of intraoperative hypotension with the use of the Hypotension Prediction Index and our hemodynamic management protocol. We also observed an increase in hypertensive episodes in this group but an analysis in the average hypertension between the two groups showed only a minor difference (approximately 2 mmHg) reaching marginal statistical significance. We also observed an increase in vasopressor (phenylephrine) use adjusted by patients’ weight, in the HPI group.

Our results are in agreement with a similar, previously published, preliminary study by Wingberge et al. [18] involving 60 patients. They observed a significant decrease in time weighted average of intraoperative hypotension (0.10 vs. 0.44, *p* < 0.001) with the use of an HPI-based hemodynamic diagnostic guidance and treatment protocol without however reporting an increase in the number of hypertensive episodes nor in vasopressor use, as was observed in our study. Likewise, a study of 50 patients undergoing primary total hip arthroplasty under general anesthesia [19] observed a significant reduction of 40% in intraoperative hypotension when the Hypotension Prediction Index was used for hemodynamic treatment. It is probable that the overtreatment of hypotension observed in this study was due to increased sensitivity of the clinicians involved to the early prediction system. Thus, it cannot be excluded that several alarms of the HPI system were derived from surgical manipulation that was promptly resolved, leading to initiation of hemodynamic interventions by the anesthesiologist in charge and therefore to the occurrence of hypertension and increased vasopressor use. Nonetheless, the occurrence of intraoperative hypertension is not associated with any of the adverse outcomes associated with hypotension such as 30-day mortality as reported by Monk et al. in a retrospective analysis of more than 18,000 patients [7]. Judicious use of this new prediction model is warranted to avoid cases of inappropriate use (e.g., false positive alarms) that could potentially lead to overtreatment.

A prospective randomized trial by Maheshwari et al. [20], surprisingly, did not find any difference in the incidence of intraoperative hypotension between 214 HPI-guided and unguided patients undergoing non cardiac surgery. A possible explanation of the findings in that study includes aggressive treatment of hypotension in the control group (e.g., Hawthorne effect), which is reasonable given the low incidence of intraoperative hypotension in both groups (TWA of IOH: 0.14 min average). The authors explicitly state in the manuscript that they instructed clinicians to avoid hypotension in both groups. Other causes might include the complexity of the index algorithm, lack of familiarity for novel technology, and lack of clinicians’ responses to the alert. In fact, more than half of the alarms were not followed by treatment in this study. Conversely, when the clinicians intervened, hypotension was reduced by 57%. It is intuitive that only adherence to treatment and changes in therapy will successfully prevent hypotension not the prediction of hypotension alone. In our study, all the anesthesiologists involved followed the treatment algorithm closely, with a reported 91% adherence rate, resulting in a marked hypotension reduction. In fact, maybe the strict adherence to the algorithm lead to more hypertension observed in the HPI group.

Our study has several limitations. Given that the study design aimed to detect only reduction of intraoperative hypotension, we were not able to detect any significant differences in clinical outcomes such as cardiac or renal complications or reduction in mortality with the use of this predictive index. Moreover, a “Hawthorne effect” cannot be excluded since all the physicians involved were aware of the participation in the trial. Knowing that the endpoint of the trial was to reduce IOH might have influenced all physicians involved in the trial, both in the intervention and control groups, to improve their performance regarding treatment of hypotension. However, the incidence of hypotension in the control group was similar to the degree of hypotension measured in our hospital prior to initiation of the trial (TWA-MAP: 0.46 mmHg vs. 0.54 mmHg), therefore a significant bias from participation in the trial could not be detected. Third, this was, similarly to previous trials, a single center study wherein we excluded patients with certain cardiac conditions, therefore the generalizability of our results might be limited. Evaluation of the HPI in different clinical conditions and patients’ population is required. In the future, multicenter trials aimed at detecting clinical outcomes and not physiological parameters are warranted in order to affirm the usefulness of this prediction model. Fourth, the accuracy of pulse contour analysis methods to evaluate cardiac index and stroke volume has been debated [21,22,23]. This should be taken into account when planning a hemodynamic management protocol based on these devices. Finally, the Hypotension Prediction Index was used along with a treatment protocol conceived in our center, which is not validated, but derived from clinical experience and existing literature. Hence it is possible that this treatment protocol might be improved in the near future.

In conclusion, in this single center prospective study, we demonstrated that the use of the Hypotension Prediction Index integrated in a hemodynamic treatment protocol is useful in predicting and preventing intraoperative hypotension. We also observed an increase in the time spent in hypertension in the treatment group, probably as a result of overtreatment. This should provide insight for refining the use of this prediction index in future studies avoiding excessive correction of blood pressure.

## Figures and Tables

**Table 1:** Baseline Characteristics.

Characteristics	HPI (*n* = 49)	Control (*n* = 50)
Age, median (IQR), yr	66 (58–74)	70 (57–75)
Sex, n (%)		
Male	26 (53%)	29 (58%)
Female	23 (47%)	21 (42%)
BMI, mean ± SD, kg/m^2^	27.7 ± 5	27.4 ± 4.5
Previous medical history, n (%)		
Cardiovascular diseases	6 (12.3%)	1 (2%)
Hypertension	26 (53%)	21 (42%)
Pulmonary Diseases	6 (12.5%)	5 (10%)
Diabetes	6 (12.5%)	14 (28%)
Metabolic Diseases	8 (16.3%)	5 (10%)
Liver Disease	3 (6.1%)	1 (2%)
Obesity/Weight loss	5 (10.2%)	2 (4%)
Preoperative tests, mean ± SD		
Hemoglobin (g/dL)	12.7 ± 2.1	12.1 ± 2
MAP (mmHg)	97 ± 14	93 ± 19
Creatinine (mg/dL)	0.87 ± 0.46	0.81 ± 0.22
eGFR (mL/min/1.73 m^2^) *	97 ± 33	94 ± 30
ASA class, n (%)		
I	3 (6%)	3 (6%)
II	39 (80%)	42 (84%)
III	7 (14%)	5 (10%)
Type of Surgery, n (%)		
General	6 (12%)	7 (14%)
Gastrointestinal	20 (41%)	19 (38%)
Hepatobiliary	15 (31%)	14 (28%)
Orthopedic	3 (6%)	4 (8%)
Neurosurgical	1 (2%)	4 (8%)
Vascular	3 (6%)	0 (0%)
Maxillofacial	1 (2%)	2 (4%)
Baseline SVI, median (IQR), (mL/m^2^)	40 (31–52)	37 (31–46)
Duration, median (IQR), (min)		
Anesthesia	240 (187–280)	240 (182–404)
Surgery	207 (150–255)	207 (150–332)

Abbreviations: BMI, body mass index; MAP, mean arterial pressure; eGFR, glomerular filtration rate; ASA, American Society of Anesthesiology; SVI, stroke Volume index. * calculated using the MDRD 4-variable formula GFR (mL/min/1.73 m^2^) = 175 × (Scr) − 1.154 × (Age) − 0.203 × (0.742 if female) × (1.212 if African American) (conventional units).

**Table 2:** Outcomes.

Endpoints	HPI (*n* = 49) Median (IQR)	Control (*n* = 50) Median (IQR)	Median Difference (95% CI)	*p* Value
Hypotension *				
TWA hypotension, mmHg	0.16 (0.01–0.32)	0.50 (0.11–0.97)	−0.28 (−0.48 to −0.09)	0.0003
Area Under the Threshold, mmHg * min	42.42 (2.07–100.86)	104.71 (28.02–351.54)	−60.28 (−111.09 to −17.71)	0.0012
Number of hypotensive events, *n*	26 (6–54)	73 (32–190)	−43 (−78 to −18)	0.0002
Time in Hypotension, min	8.68 (2.0–17.3)	24.16 (10.7–63.)	−14.33 (−26 to −6)	0.0002
Percentage of Hypotension, %	3.7 (0.89–6.31)	10.17 (3.41–16.79)	−6.67 (−9.57 to −3.41)	<0.0001
*Hypertension* ^$^				
TWA hypertension, mmHg	0.95 (0.27–1.81)	0.29 (0.08–0.83)	0.40 (0.10 to 0.83)	0.0032
Area Above the Threshold, mmHg * min	245.49 (94.36–393.04)	82.10 (25.62–174.51)	99.88 (29.10 to 196.33)	0.0032
Number of hypertensive events, *n*	73 (40–115)	34 (14–65)	29 (11 to 51)	0.0022
Time in Hypertension, min	24.30 (13.3–38.)	11.33 (4.7–21.7)	9.67 (3.6 to 16.7)	0.0024
Percentage of Hypertension, %	9.71 (3.87–18.05)	4.09 (1.79–9.44)	3.85 (1.23 to 7.32)	0.0034
Average Hypertension Above 100	109.33 (105.46–112.39)	106.91 (103.63–110.75)	2.09 (0.01 to 4.43)	0.0438

* hypotension was defined as MAP < 65 mmHg for 1 min; ^$^ hypertension was defined as MAP >100 mmHg for 1 min; TWA = time-weighted average; HPI = Hypotension Prediction Index.

**Table 3:** Intraoperative drug use and postoperative data.

Intraoperative Data			
Medications, Median (IQR)	HPI (*n* = 49)	Control (*n* = 50)	*p* Value
Crystalloids (L)	2.5 (2–4.6)	2.7 (2.13–4.45)	0.656
Colloids (mL)	0 (0–500)	0 (0–375)	0.53
Phenylephrine (mg)	6.5 (2.4–11.3)	3.8 (0.65–9.8)	0.135
Phenylephrine adjusted for weight (mg/kg)	0.43 (0.16–0.67)	0.27 (0.06–0.6)	0.029
Ephedrine (mg)	5 (0–17.5)	7.5 (0–17.5)	0.275
Propofol (mg)	180 (150–240)	200 (170–250)	0.289
Remifentanil (mg)	0 (0–0)	0 (0–0.55)	0.964
Fentanyl (mg)	0.2 (0.15–0.3)	0.25 (0.15–0.4)	0.512
Morphine (mg)	0 (0–0)	0 (0–6)	0.483
Epidural analgesia, *n* (%)	29 (59%)	27 (54%)	0.603
Urine output (mL)	600 (400–1100)	700 (255–1150)	0.807
Blood loss (mL)	350 (200–500)	500 (200–500)	0.108
**Postoperative data**			
Length of stay in hospital (days)	10 (6–14)	11.5 (6–15.25)	0.539
Complications, *n*			
*Cardiac*			0.850
Atrial Fibrillation	5	5	
Left ventricular Failure	0	0	
Myocardial Ischemia *	1	2	
*Pulmonary*			0.310
Pneumonia	1	0	
Pneumothorax	0	0	
ARDS	0	0	
*Renal*			0.505
AKIN stage 1	4	5	
AKIN stage 2	2	1	
Surgical			0.988
Intestinal ischemia	0	1	
Portal vein thrombosis	1	0	
Mortality	1	1	0.988

* myocardial ischemia was detected as an elevation of Troponin T plasma levels.

## Data Availability

The data presented in this study are available on request from the corresponding author.
